# Development of a zoonotic influenza distribution assessment and ranking system (ZIDAR): Technical application in Nepal to support cross-sectoral risk-based surveillance

**DOI:** 10.1016/j.onehlt.2025.100975

**Published:** 2025-01-13

**Authors:** Adam Charette-Castonguay, Dipendra Gautam, Binay Shrestha, Hemant Chandra Ojha, Barun Kumar Sharma, Mukul Upadhayaya, Sujan Rana, Roshika Shrestha, Lok Bandu Chaudhary, Bhawana Kandel, Rudra Prasad Marasini, Sharmila Chapagain, Tulsi Ram Gompo, Surendra Karki, Apsara Poudel, Saugat Shrestha, Avinash Sunny Kayastha, Arun Kumar Govindakarnavar, Reuben Samuel, Allison Gocotano, Pushpa Ranjan Wijesinghe, Nilesh Buddha, Edwin Ceniza Salvador, Manish Kakkar, Ricardo J. Soares Magalhães

**Affiliations:** a Queensland Alliance for One Health Sciences, School of Veterinary Sciences, The University of Queensland, Gatton 4343, Australia; b CSIRO Agriculture & Food, Commonwealth Scientific and Industrial Research Organisation, St Lucia 4067, Australia; c WHO Country Office for Nepal, Kathmandu, Nepal; d Department of Health Services, Ministry of Health and Population, Kathmandu, Nepal; e Department of Livestock Services, Ministry of Agricultural Development, Kathmandu, Nepal; f Food and Agriculture Organization of the United Nations, Nepal; g Forest Research and Training Centre, Ministry of Forest and Environment, Nepal; h WHO Health Emergencies Department, WHO South-East Asian Regional Office, New Delhi, India; i Children's Health and Environment Program, UQ Children's Health Research Centre, The University of Queensland, Australia

**Keywords:** Zoonoses, Influenza, Mapping, Decision-support, Modelling

## Abstract

Zoonotic influenza poses a significant public health concern to agricultural industries, food security, wildlife conservation, and human health. Nations situated along migratory bird flyways and characterised by dense populations of livestock and humans, and low biosecurity of production animal value chains are particularly vulnerable to zoonotic influenza outbreaks. While spatial risk assessments have been used to map vulnerable areas, their applicability across multiple sectors has been so far limited. Here, we introduce the development and application of a Zoonotic Influenza Distribution and Ranking (ZIDAR) framework to identify areas highly suitable for zoonotic influenza transmission across multiple exposure interfaces and to measure the importance of associated risk factors. The development of ZIDAR involves a seven-step approach distributed across an initial expert consultation stage followed by a technical modelling stage. The expert consultation stage aims to define interfaces of exposure across human, livestock and wildlife, identification of associated risk factors for each of the identified interfaces and a prioritisation activity to define weights for the interfaces and associated risk factors. This is then followed by a technical phase involving model building, model structure validation, data gathering and assessment of model performance. The model development and performance assessment steps of the technical stage includes a model calibration step to maximise model fitness with regards to wildlife and animal interfaces by finding pareto-efficient sets of weights for risk factors. We applied the ZIDAR framework in Nepal and the resulting model structure enabled the identification of hotspot areas where the risk of transmission is more significant across multiple interfaces simultaneously. The ZIDAR Nepal model's predictive accuracy, determined by the area under the receiver operating characteristic curve, demonstrated strong performance: 0.87 and 0.85 for the wildlife and animal components, respectively. The ZIDAR framework presented here provides valuable insights to enable the formulation of comprehensive One Health surveillance programs and inform targeted and effective interventions to bolster pandemic preparedness strategies.

## 1 Introduction

Zoonotic influenza A viruses pose significant risks to human and animal health and the global economy. The ability of influenza A to mutate and potentially transmit from wild to domesticated birds and humans raises the spectre of severe outbreaks and pandemics, resulting in respiratory illnesses and fatalities [1,2]. In terms of economic impacts, avian influenza can lead to increased healthcare costs, severely disrupt the agricultural sector with culling of poultry flocks and trade restrictions on animal products, and cause supply chain disruptions, leading to shortages and price fluctuations in various goods [3,4]. Proactive measures such as early detection, surveillance, and vaccination programs are crucial to mitigate these risks and prevent widespread health and economic losses. To effectively prepare for and prevent this threat, decision-makers must be able to identify the main mechanisms of transmission and in which geographical areas outbreaks are more likely to occur.

Several studies have investigated spatial risk of zoonotic influenza transmission in wild birds and poultry. For instance, species distribution modelling was utilised to simulate spatial risk profiles of H7N9 and H5N1 virus subtypes across mainland China [5]. In areas where reported zoonotic influenza data are abundant, spatial regression models have been developed linking reported highly pathogenic avian influenza (HPAI) H5N1 and risk factors including elevation, human population, chicken numbers, duck numbers, and rice cropping intensity [6,7,8]. In geographical areas where detections are more scarce, spatial multicriteria decision analysis (MCDA) models have been developed to produce suitability maps for HPAI H5N1 and low pathogenicity avian influenza in poultry [9,10,11]. In the context of suitability mapping the term “suitability” refers to the degree to which a specific geographic area is appropriate or favourable for a particular purpose or activity, which in this case is exposure to zoonotic influenza. Suitability is determined by evaluating a variety of factors by creating spatial representations to highlight areas that meet the defined criteria for suitability. Focussing specifically on wildlife interfaces, Prosser et al. [12] assessed the spatial risk factors for HPAI H5N1 transmission from winter bird risk in China.

Previous studies have provided several geospatial modelling platforms for the identification of geographical vulnerabilities for single sectors and in single hosts limiting their applicability for integrated surveillance decisions. For example, in most studies waterfowl density was considered as a risk factor, but the epidemiological role of this factor on the transmission risk was not differentiated for wildlife, poultry and human populations. Furthermore, there is a lack of studies aiming to develop a harmonised platform of data elicitation for the identification of human communities at risk of exposure that is bespoke to the context of countries of application. To our knowledge, no studies have involved end-users of models in the model structure co-design process nor developed model structures to allow spatial risk assessment across multiple interfaces of exposure such as wild birds, animal production or humans.

To address this gap in how maps can be utilised for programmatic country-level decisions across sectors affected by zoonotic influenza, we developed a geospatial operational framework to provide guidance to countries on localities where, under a certain set of criteria, suitability for zoonotic influenza exposure is highest across a range of potential interfaces. The importance of a One Health bottom-up approach with regards to the development of operational surveillance tools in the context of zoonotic disease threats has been highlighted in previous studies [13,14]. This means that to support decisions locally and enable utilisation and adoption of maps as decision-support tools for zoonotic disease surveillance, stakeholders need to be involved in the process of model development and validation from the beginning. One way this can be achieved is through the process of expert consultation to identify key interfaces of zoonotic influenza exposure, associated risk factors that are locally important to be considered and estimated. Another way is through the process of involving local experts across sectors in the process of model building and validation. This not only allows to foster trust leading to sustainable model utilisation and adoption, but also provides essential local capacity building of in-country surveillance and epidemiology teams. Country utilisation of model outputs is further enhanced through the development of a spatial decision support system (SDSS) that includes a web-based user interface to facilitate the interactive exploration and visualisation of areas where the release and exposure to zoonotic influenzas across human and animal hosts is highest.

In this paper, we present the process and results of development of the ZIDAR framework in Nepal, which is a country considered to be at high risk of zoonotic influenza outbreaks given its location on the Central Asian Flyway [15,16,17], the presence of live bird markets [18] and the large proportion (close to 35 %) of population participation in agriculture [19]. The aim of the study was to identify hotspots in Nepal where the suitability for zoonotic influenza exposure across multiple sectors is highest and introduce ZIDAR as a generalisable tool to support local surveillance decisions across sectors for other high-risk countries.

## 2 Methods

**Fig. 1:**
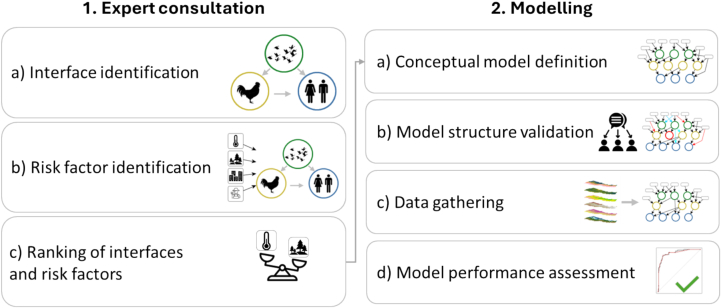
Graphical representation of the steps involved in the expert consultation process and the model co-development and assessment step.

The development of the ZIDAR framework involves an expert consultation stage (comprised of 3 steps) and a technical modelling stage (comprising 4 steps). The expert consultation stage aims to: a) identify interfaces of exposure across human, livestock and wildlife, b) identify associated risk factors for each interface and c) conduct a prioritisation activity to define weights for the interfaces and associated risk factors. The technical phase involves four steps: a) conceptual model development, b) model structure validation, c) data gathering and d) assessment of model performance (Fig. 1).

The expert elicitation process is an important first step to inform the development of the three ZIDAR sub-models in the technical phase: 1) wildlife zoonotic influenza distribution and ranking model (ZIDAR-W), 2) animal zoonotic influenza distribution and ranking model (ZIDAR-A) and 3) human zoonotic influenza distribution and ranking model (ZIDAR-H). ZIDAR-W aims to assess the spatial variation in the suitability of wildlife exposure to zoonotic influenza, specifically wild birds. ZIDAR-A enables the assessment of the spatial variation in the suitability for farmed animal exposure to zoonotic influenza, including poultry and swine production, and retail interfaces such as live animal markets. ZIDAR-H assesses the spatial variation in the suitability of human exposure to zoonotic influenza. Interfaces of exposure included in ZIDAR-W, −A and -H were discussed in consultation with national technical experts from animal, human and environmental sectors during the workshop.

### 2.1 Conceptualising a three-tiered model of zoonotic influenza transmission

The first step of the expert consultation stage involves the elicitation of country-level expertise to identify the most important interfaces of exposure to zoonotic influenza across different hosts relevant to the country. To that effect, experts involving human health, animal health and environmental health are gathered for a workshop to identify: 1) the most relevant interfaces of exposure to zoonotic influenza for human, animal, and wildlife populations, and 2) the key risk factors that would modulate the exposure risk for each of the hosts in each of the identified interfaces in the country of interest. For a detailed example of the application of this process to the context of Nepal please see Appendix. In addition to identifying key interfaces and risk factors, the workshop aimed to, estimate the relative importance of risk factors, co-design the model structure and identify data sources to inform risk factors and parameterise the model.

Following on from the expert elicitation on the critical interfaces of exposure to zoonotic influenza, experts are consulted to consider key risk factors for each interface of exposure identified. This step formed the co-design of the conceptual model to map interfaces of transmission and risk factors across wildlife, animal and human interfaces organised as a directed acyclic graph (DAG). Using this DAG framework, interfaces are represented as latent constructs, which can only be inferred indirectly through a mathematical model, and risk factors are represented as measured variables. Each group of hosts, i.e., wildlife, farmed animals and humans, can be composed of multiple interfaces, e.g., live animal markets and backyard poultry farming interfaces in ZIDAR-A. In the case of ZIDAR Nepal, the structure of the conceptual model including interfaces of transmission and risk factors was reviewed and further developed by local experts during a subsequent stakeholder engagement activity. This step serves as a qualitative validation of the conceptual model and DAG.

### 2.2 Ranking of risk factors and interfaces

Not all interfaces and associated risk factors for zoonotic influenza identified in the previous step are equally important for transmission to occur between and within hosts. The third part of the workshop aims to elicit from experts the relative importance of each interface within a particular host type and rank the relative importance of the risk factors within the identified interfaces.

The process of expert elicitation of the relative importance of interfaces and factors involves pair-wise assessment of importance using the Analytic Hierarchical Process (AHP) [20]. Experts are asked to compare the relative importance of interfaces and factors within an interface on a qualitative scale ranging from 1/9 representing *Extremely less important*, to 9 representing *Extremely more important*. The consistency of an expert's pairwise comparison matrix should be assessed using the consistency ratio. A pairwise comparison matrix is considered sufficiently consistent when the consistency ratio is lower than 0.10 [20]. Interfaces and risk factors of ZIDAR-H, ZIDAR-A and ZIDAR-W are weighted by the relevant One Health experts, i.e., human health, animal health and environmental health experts. The resulting relative importance of each interface and risk factor can then be used to parameterise the model.

### 2.3 Model calibration and validation

We use a multicriteria decision analysis model to estimate suitability of exposure in each interface. We first start by estimating the suitability of each interface using a weighted sum of risk factors and associated weights:

$${\mathrm{IS}}_{\mathrm{ik}}=\sum_{j=1}^{n}w_{\mathrm{jk}}{\mathrm{RF}}_{\mathrm{ijk}}$$

where *IS*_*ik*_ is the suitability of an interface *k* estimated for each raster cell *i*, *w*_*jk*_ is the weight for risk factor *j*, *RF*_*ij*_ is the value of risk factor *j* for raster cell *i* and *n* is the number of risk factors for an interface. We then calculated the aggregated suitability of each combined interfaces, i.e., total wildlife, animal, and human suitability, using a weighted sum of individual interfaces and associated weights:

$${\mathrm{TS}}_{i}=\sum_{k=1}^{m}w_{k}{\mathrm{IS}}_{\mathrm{ik}}$$

where *TS*_*i*_ is the total suitability across all interfaces of a host (e.g., total wildlife suitability) estimated for each raster cell *i*, *w*_*k*_ is the weight attributed to interface *k*, and *m* is the number of interfaces in each group of hosts.

The model was then coded using R programming language and is parameterised using the mean weight of each interface and risk factor across all participants' responses. Each risk factor in the model is a different spatial layer. In the context of Nepal, the datasets for the risk factors identified during the workshop were obtained through different sources (Appendix Table 1) and all spatial layers were converted to raster maps at 10 km spatial resolution to align with the resolution of livestock density and annual occurrence of migratory bird.

Validation of the model is conducted in two steps: 1) qualitative validation of the conceptual model with local experts (described above) and 2) quantitative validation of model results using the area under the curve (AUC) of the receiver operating characteristic (ROC) curve metric for as many interfaces as possible. The latter is performed by considering the available reported number and location of observed zoonotic influenza cases in specific interfaces of exposure. In the case of Nepal, this validation could be conducted for the wildlife (ZIDAR-W) and the animal (ZIDAR-A) sub-models. The AUC ROC metric was estimated by comparing the ZIDAR-W and ZIDAR-A suitability maps with cases of influenza (H5N1 and H5N8) reported to date in wild birds (crows, whooper swan and Asian openbill; seven cases in total) and poultry (213 cases of H5N1) across Nepal, respectively. Reported cases for validation were obtained from the WAHIS [21] database and divided among wildlife and poultry cases. The absence of information on the geo-location of reported human cases of avian influenza did not allow to validate the ZIDAR-H sub-model output map.

Since the relative importance of risk factors and interfaces vary based on expert opinions, the model can be further calibrated by sampling weight values from uniform distributions between the minimum and maximum values of each risk factor and interface resulting from the experts' weighing procedure (minimum and maximum value columns in Appendix Table 2 and Appendix Table 3). In the Nepal case study, we ran Monte Carlo simulations with 10,000 iterations and recorded sampled value along the parameter space of the weights and the associated AUC ROC of ZIDAR-W and ZIDAR-A at each iteration. Once all iterations were completed, we conducted a bi-objective optimisation to identify the weight configurations for which both AUC ROC of ZIDAR-W and ZIDAR-A were maximized. The optimal set of weights forming the Pareto frontier was obtained using rPref package in R which is based on the skyline operator [22]. This approach identifies all combinations that are not dominated by any other combinations. A combination dominates another if it is at least as good in both dimensions and better in at least one dimension. This technique allows the extraction of the pareto-efficient sets of weights, i.e., the sets of weights for which an improvement in one objective (e.g., improving the AUC of wildlife suitability) can only be achieved at the detriment of the other objective (e.g., improving the AUC of animal suitability). This approach was aimed to calibrate the model in a way that maximises the model fitness for both wildlife and poultry interfaces simultaneously.

### 2.4 Uncertainty and sensitivity analysis

Given the potentially large number of interfaces and risk factors constituting the model (18 and 19 in the Nepal application, respectively), the combination of structural and parameter uncertainties are likely to increase the uncertainty in the results of the model. Therefore, we investigated model uncertainty resulting from Monte Carlo simulations described in the previous steps. The uncertainty investigation was two-fold: 1) we assessed the variability in the fitness of the model, specifically looking at the range of AUC values for ZIDAR-W and ZIDAR-A resulting from using different weight values, and 2) we evaluated spatial uncertainty by calculating the coefficient of variation of both ZIDAR-W and ZIDAR-A suitability for each grid cell across the 1000 simulations.

In addition to uncertainty assessment, we also evaluated the sensitivity of the AUC ROC metric to different values of weights in the model. To do so, we generated scatter plots for each risk factor and interface to investigate how the output variables, in this case the AUC ROC of ZIDAR-W and ZIDAR-A, vary as a function of the change in each input, i.e., the weights on risk factors and interfaces.

To investigate and quantify the importance of interfaces and risk factors on exposure to zoonotic influenza, we analysed how the AUC metric varied within the simulated parameter space of the weights provided by experts for each interface and risk factor. Given the availability of validation data for wildlife and farmed animals we were able to conduct this analysis for only ZIDAR-W and ZIDAR-A.

### 2.5 Hotspot mapping

To identify areas of high suitability of both wildlife and animal transmission, we mapped hotspots of zoonotic influenza suitability across the two interfaces by overlaying suitability maps of ZIDAR-W and ZIDAR-A. We used the Fisher-Jenks classification algorithm [23] with the classInt R package [24] to classify suitability of each pixel for both ZIDAR-W and ZIDAR-A and combined classes of suitability for the two interfaces. The Fisher-Jenks classification is widely used in thematic mapping for categorising spatial values including disease risk mapping [25]. The algorithm facilitates visual interpretation of spatial data by optimally classifying observations into classes such that inter-class variance is maximized, and intra-class variance is minimized [26]. We then mapped hotspots of two-dimensional transmission suitability with bi-variate choropleth.

## 3 Results

The first expert consultation workshop in Nepal identified a total of 18 interfaces of zoonotic influenza exposure across wildlife (ZIDAR-W), animal (ZIDAR-A) and human interfaces (ZIDAR-H). Animal interfaces were further categorised into poultry (ZIDAR-P), swine (ZIDAR-S) and mixed production systems.

### 3.1 Exposure suitability in wildlife interfaces

**Fig. 2:**
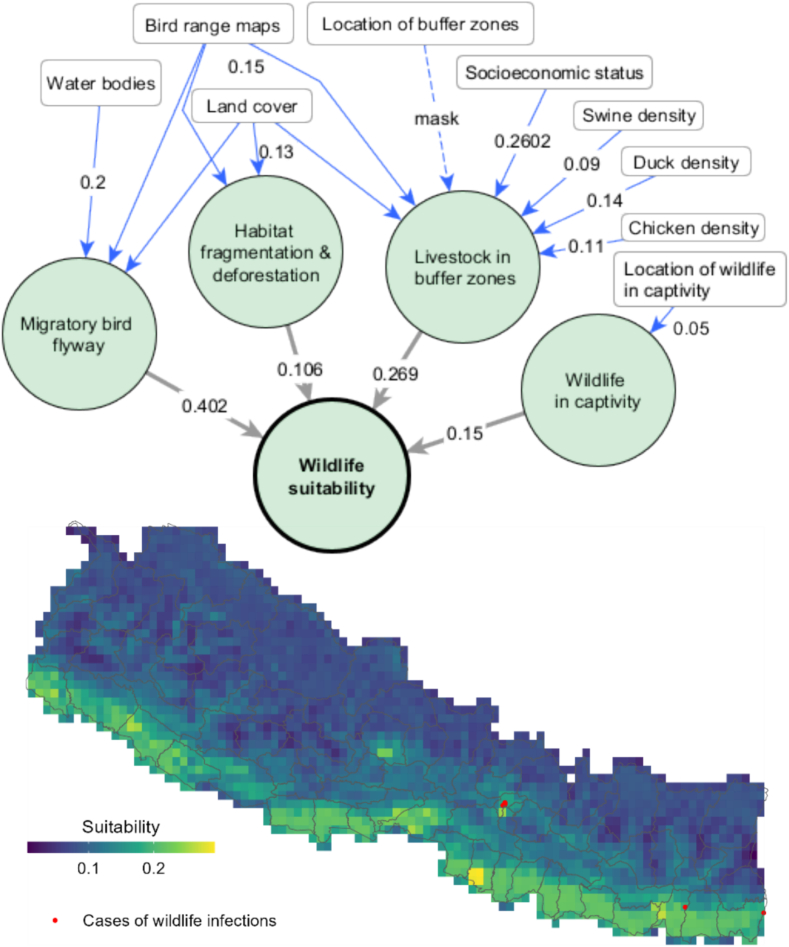
Interfaces and risk factors of wildlife transmission suitability. Blue lines correspond to the weighted influence of a risk factor over an interface. Grey lines correspond to the relative importance of each interface towards the total wildlife suitability. The map corresponds to the simulated suitability of zoonotic influenza in wildlife in comparison to reported cases of wild bird infections. (For interpretation of the references to colour in this figure legend, the reader is referred to the web version of this article.)

The final zoonotic influenza suitability model for wild bird populations (ZIDAR-W) considered a total of four interfaces of zoonotic influenza transmission for wildlife species. National experts indicated that the main interfaces for wild bird exposure (depicted as green circles in Fig. 2) are locations within migratory bird flyways, landscapes classified with habitat fragmentation and deforestation, wildlife buffer zones containing livestock and wildlife captivity settings such as zoos.

A total of nine risk factors (depicted as white rectangular boxes in Fig. 2) were identified as influencing exposure of wild birds in these interfaces. The blue lines correspond to the weighted influence of a risk factor over an interface and the grey lines correspond to the relative importance of each interface towards the total exposure suitability of wild birds to zoonotic influenza. The resulting suitability maps indicate that total exposure suitability of wild birds to zoonotic influenza (Fig. 2).

The final wildlife suitability map shows higher suitability for exposure in the southern districts of the country and around urban centres Kathmandu and Pokhara due to higher habitat fragmentation and greater density of nesting birds. Suitability maps of each specific sub-interfaces are displayed in Appendix 4. Model validation for ZIDAR-W was conducted using the AUC ROC metric by comparing total wildlife suitability map with cases of influenza reported to date in wild birds in Nepal. The AUC ROC obtained using mean weights of interfaces (Appendix Table 2) and risk factors (Appendix Table 3) was 0.876 indicating excellent model discrimination, although more data points of observed infections will be needed in the future to enhance the reliability of the model results of total wildlife suitability.

### 3.2 Exposure suitability in animal production and retail interfaces

**Fig. 3:**
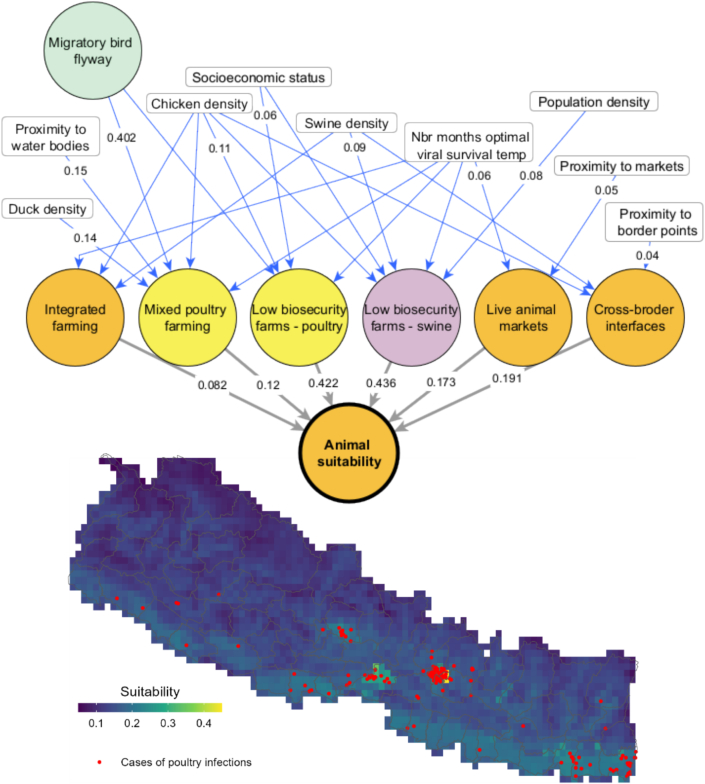
Interfaces and risk factors of animal transmission suitability. Blue lines correspond to the weighted influence of a risk factor over an interface. Grey lines correspond to the relative importance of each interface towards the total animal suitability. The map corresponds to the simulated suitability of zoonotic influenza in animal production and retail systems in comparison to reported cases of poultry infections. (For interpretation of the references to colour in this figure legend, the reader is referred to the web version of this article.)

The final map of influenza exposure suitability for animal production and retail (ZIDAR-A) considered sub-models for poultry (ZIDAR-P) and for swine (ZIDAR-S). In the case of ZIDAR-P a total of five interfaces of zoonotic influenza transmission for poultry species, including integrated farming systems (i.e., multi- species husbandry systems), mixed poultry farms, low biosecurity poultry farms, live animal markets and cross border interfaces (depicted as orange and yellow circles in Fig. 3). In the case of ZIDAR-S a total of four interfaces were considered including integrated farming systems (i.e., multi-species husbandry systems), low biosecurity swine farms, live animal markets and cross border interfaces (depicted as orange and purple circles in Fig. 3).

National experts identified that the exposure of livestock in these interfaces was modulated by a total of ten risk factors (depicted in white rectangular boxes in Fig. 3) including the migratory bird suitability map from ZIDAR-W. The blue lines correspond to the weighted influence of a risk factor over an interface and the grey lines correspond to the relative importance of each interface towards the total exposure suitability of farmed animal species to zoonotic influenza.

The resulting suitability maps show that total exposure suitability of farmed animals to zoonotic influenza is highest in very small clusters around Kathmandu and Bharatpur, where high poultry, human and wild bird densities intersect. Model validation for ZIDAR-A was conducted using the AUC metric by comparing total poultry suitability map (Fig. 3) to a geolocated database of 213 cases of avian influenza in poultry. The AUC of the model parameterised with mean weights of interfaces (Appendix Table 2) and risk factors (Appendix Table 3) was 0.857, indicating excellent model discrimination.

### 3.3 Exposure suitability in human interfaces

**Fig. 4:**
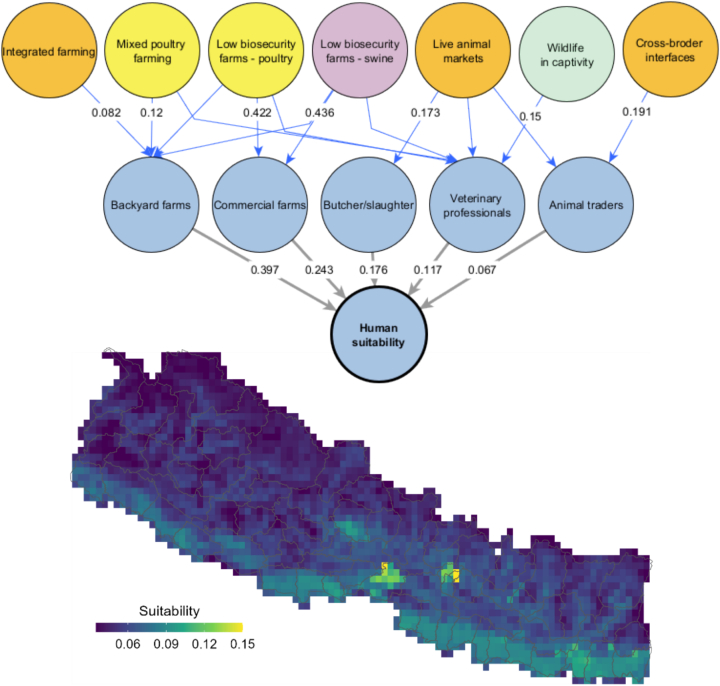
Interfaces and risk factors of human transmission suitability (blue circles). Blue lines correspond to the weighted influence of a risk factor (here, animal and wildlife interfaces) over a human interface. Grey lines correspond to the relative importance of each interface towards the total human suitability. (For interpretation of the references to colour in this figure legend, the reader is referred to the web version of this article.)

The final zoonotic influenza suitability map for human populations (ZIDAR-H) considered a total of five interfaces of zoonotic influenza transmission for humans. National experts indicated that the main interfaces for human exposure (depicted as blue circles in Fig. 4) included locations with backyard farms, commercial farms, and locations likely to have a higher density of butchers and slaughters, veterinary professionals and animal traders. National experts identified a total of seven risk factors, which were the key interfaces of ZIDAR-P and ZIDAR-S and captivity settings with wild birds from ZIDAR-W (depicted in orange, yellow, purple and green circles in Fig. 4). The blue lines correspond to the weighted influence of animal and wildlife interfaces and the grey lines correspond to the relative importance of each human interface towards the total exposure suitability of humans to zoonotic influenza. The resulting suitability maps indicate that total exposure suitability of humans to zoonotic influenza is highest in small clusters in densely populated areas (e.g., Kathmandu valley, Pokhara and Bharatpur) and along the southern border and the southeast region of the country. In this case, no geo-located database of reported human infection was available for comparison.

### 3.4 Hotspots for wildlife and animal transmission

**Fig. 5:**
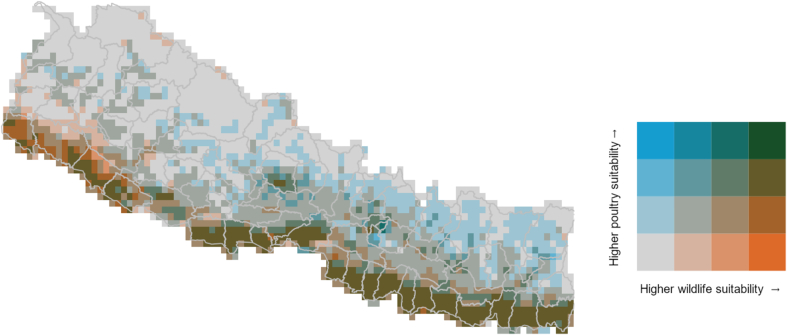
Hotpots mapping of zoonotic influenza exposure suitability in wildlife and animal interfaces in Nepal.

The hotspot map highlights highly suitable areas for both interfaces in the south central and eastern regions of the country, as well as in urbanised areas such as Kathmandu and Pokhara (Fig. 5). The western part of the country shows high suitability for wildlife transmission, albeit lower suitability for animal interfaces (orange pixels in Fig. 5). Conversely, the Central eastern part of the country shows areas of high suitability for poultry transmission, however given the distance to nesting and wintering sites, these areas have lower suitability for wild bird exposure than the southern part of the country (blue pixels in Fig. 5).

### 3.5 Optimisation of model calibration

**Fig. 6:**
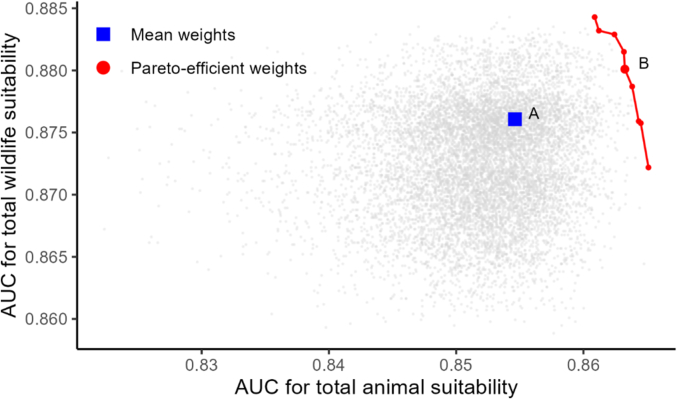
Fitness of the model characterised by the AUC of wildlife suitability (vertical axis) and AUC of animal suitability (horizontal axis) from 10,000 simulation runs with different sets of weights. The blue square (A) indicates the result of the model with the mean weights of risk factors and interfaces, whereas red dots show model runs with pareto efficient sets of weights. Dot B shows one of the optimal set of weights used to produce maps in Fig. 7. (For interpretation of the references to colour in this figure legend, the reader is referred to the web version of this article.)

In the case of the application in Nepal, optimal calibration of weights could slightly increase the fitness of both ZIDAR-W and ZIDAR-P sub-models. From the 10,000 Monte Carlo simulation runs, each grey point in Fig. 6 represents a different outcome of the model calibrated with a specific set of weights with an associated model fitness (AUC) for ZIDAR-W (vertical axis) and ZIDAR-P sub-models (horizontal axis). From all simulations, we identified the pareto frontier, which includes 9 pareto-efficient model calibrations highlighted as red dots in Fig. 6. For instance, using a pareto-efficient set of weights illustrated as point B in Fig. 6 instead of mean weights (point A in Fig. 6) increases the AUC from 0.876 to 0.88 and 0.857 to 0.863 for ZIDAR-W and ZIDAR-P sub-models, respectively.

**Fig. 7:**
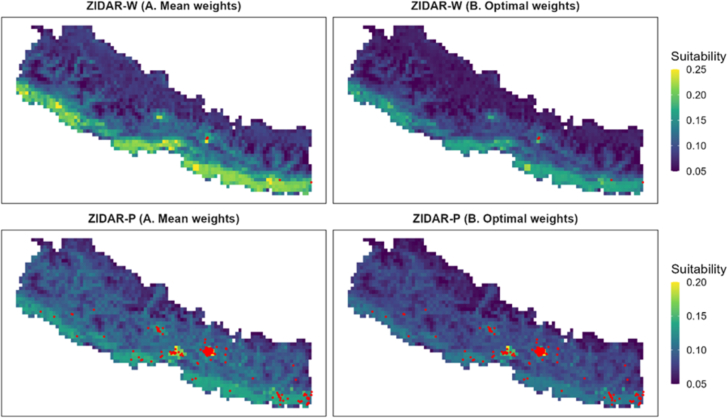
Suitability maps for ZIDAR-W (top panels) and ZIDAR-P (bottom panels) shown for simulation using mean weights (left panels, corresponding to square A in Fig. 6) and using optimal set of weights (right panels, corresponding to point B in Fig. 6). Wildlife (top panels) and animal reported cases (bottom panels) are shown in red. (For interpretation of the references to colour in this figure legend, the reader is referred to the web version of this article.)

Based on the results of this calibration, we used the pareto-efficient weights and developed optimised maps of wild bird (Fig. 7, top panels) and poultry (Fig. 7, bottom panels) zoonotic influenza exposure suitability. Compared to initial un-optimised maps (left panels in Fig. 7) the ZIDAR-W and ZIDAR-P optimised maps (right panels in Fig. 7) demonstrate high degree of suitability clustering with very clear and circumscribed clusters and lower suitability in areas where no cases have been reported, e.g., western part of the country.

### 3.6 Uncertainty and sensitivity analysis

**Fig. A1:**
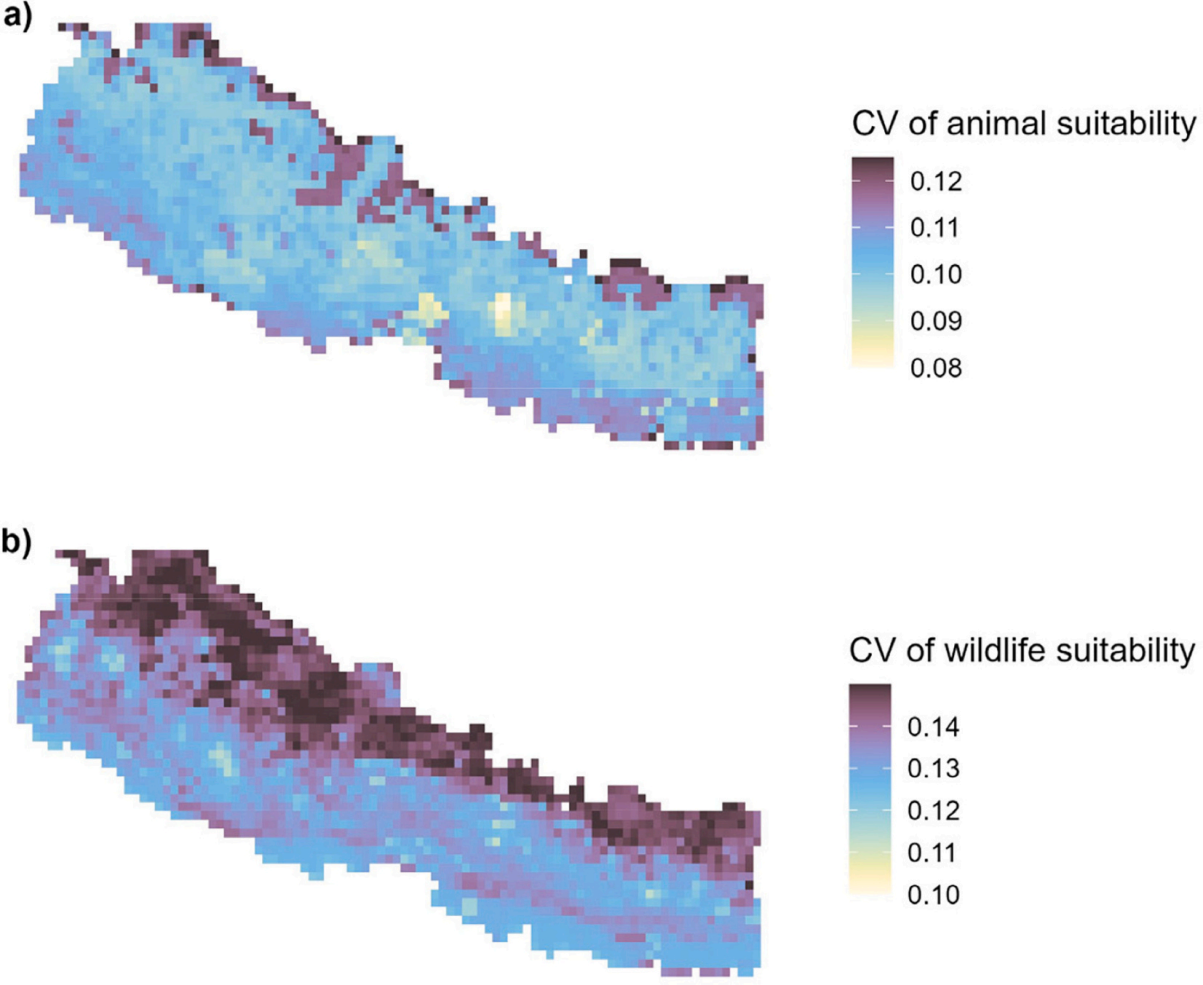
Spatial uncertainty of a) animal suitability sub-model and b) wildlife suitability sub-model.

The model shows robustness in its goodness-of-fit with 95 % of the 10,000 simulations resulting in an AUC between 0.842 and 0.859 for the animal suitability component and between 0.865 and 0.88 for the wildlife component (Fig. 6). Furthermore, model results show relatively low spatial variability demonstrated by the low coefficient of variation of suitability for both ZIDAR-W and ZIDAR-A (Appendix 5, Fig. A1). The pixels with high coefficient of variation are situated in the northern part of the country where suitability for transmission is lower, whereas the southern part of the country displays lower variability in suitability.

**Fig. A3:**
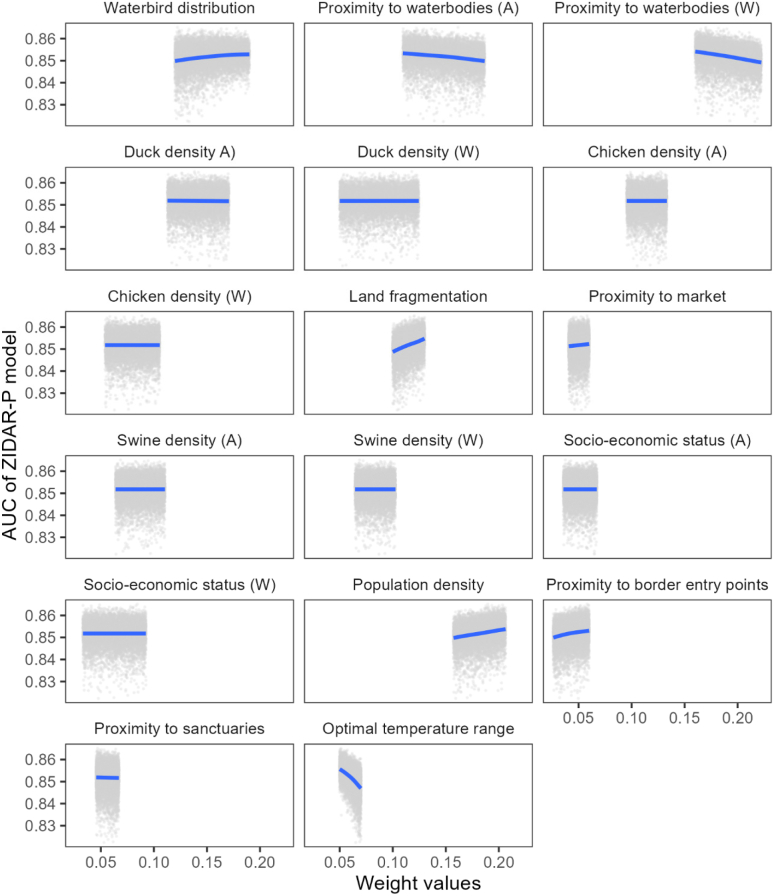
Association between the weight attributed to each risk factor and the AUC of ZIDAR-P model validation after 10,000 iterations.

Our results indicate that the interfaces of migratory bird flyways, poultry farming with low biosecurity and cross-border interfaces had the largest positive impact on improving the fitness of the model, i.e., an increase in the weight of these factors had the largest improvement in the AUC of animal suitability (Fig. A3).

Conversely, integrated farming, low biosecurity swine farming and live animal markets had the largest negative impacts on the fitness of the model when comparing total animal suitability with observed cases. This could be explained by the omission of animal cases from animal markets for the validation of the model and the calculation of the AUC. Furthermore, we did not consider observed cases of swine influenza for the validation of the model to simulate animal suitability.

In addition, the risk factors of waterbird distribution, land fragmentation and population density had the greatest positive influence on the fitness of the model to simulate animal suitability (Fig. A3). Our results also show that the risk factor optimal temperature range for viral survival had a strong negative influence on model fitness. This could indicate that an inadequate measure (average monthly temperature) was used to represent optimal temperature range for viral survival in the model.

### 3.7 Interactive visualisation platform

We developed an R Shiny application that allows decision-makers to visualise interactively the results of the ZIDAR model: https://qaohs.shinyapps.io/zidar_app/ . The application allows users to visualise maps of exposure to zoonotic influenza for all 18 interfaces identified and zoom in on areas of interest. The interactive nature of R Shiny applications enables the collaboration and engagement among local stakeholders, fosters data-driven discussions and allows decision-makers to drill down into specific interfaces and regions of the countries as opposed to using static maps. This can facilitate the development of surveillance and intervention strategies for targeted areas and sectors, for instance, cross-border movements specific districts or provinces.

## 4 Discussion

### 4.1 The need for one health decisions support systems to guide surveillance for zoonotic influenza

Surveillance systems for zoonotic influenza that aim to monitor and track the evolution of influenza viruses across hosts, benefit from innovative, end-user informed and fit-for-purpose spatial decision support systems (SDSSs). Knowledge of the characteristics of the local interplay of environmental, agricultural and socio-demographic factors influencing disease transmission can be leveraged by integrating spatial information on the risk factors and interfaces of exposure where wildlife, livestock and human populations intersect. Zoonotic influenza surveillance designs guided by local knowledge becomes more comprehensive and effective, aiding in early detection, rapid coordinated response, and proactive prevention measures to mitigate the impact of these infectious diseases on both animal and human populations.

While previous studies focused on mapping risk of zoonotic influenza in a single host using models where end-users had little or no input, we presented here a new participatory modelling spatial risk assessment framework to identify key interfaces across wildlife, livestock and human populations. This allows decision-makers to be engaged in the modelling process and to create trust in model outputs to develop sustained surveillance strategies not only in particular areas, but also within specific sectors, for instance cross-border movement or live animal markets.

Our approach also allows to complement expert opinion on the relative importance of risk factors to assess exposure in each interface with a multicriteria optimisation of interfaces and risk factors weights considering uncertainty in expert opinions. This approach helps to reduce the uncertainty related to the potentially limited knowledge of stakeholders outside of their expertise. Moreover, in the case of the model application in Nepal presented here, this approach led to an improvement in the discrimination of the model. Further, this approach enables 1) the assessment of spatial uncertainty in risk assessment maps to foster robust planning, and 2) sensitivity of model outputs to the change in parameter values to identify most salient risk factors.

As part of Nepal's national surveillance plan for HPAI, the 75 districts within the country have been divided into three categories: 20 high-risk districts, 21 medium-risk districts and 34 low-risk districts [27]. In agreement with our results, high-risk districts identified within the surveillance plan are located along the southern border of the country and around major urban areas of Kathmandu and Pokhara. Our results provide greater spatial granularity of the risk assessment, which can be further explored dynamically with the interactive user interface developed with R Shiny. Therefore, the modelling approach presented here can guide policymakers to develop more targeted surveillance programs and inform interventions for pandemic preparedness in specific districts and sectors.

### 4.2 Future improvements and next steps

Model results could be improved by obtaining additional data to enable further calibration and validation of the model. In this application of the model, geo-located reported influenza cases in human population were not available and therefore, the sub-model of human exposure (ZIDAR-H) could not be validated at this time. Accessing such information for next iterations of the model will allow a holistic assessment of suitability across the three interfaces of One Health, i.e., wildlife animal and humans. Currently, only seven reported cases of influenza in wild birds were available for validation of the wildlife interface. Obtaining more data points would also help to restrict suitable areas and increase the reliability of the results. Information on human risk factors would also help to conceptualise human interfaces. For instance, population employed in veterinary services and animal slaughter were first identified by experts as risk factors for human interfaces. However, given the lack of data availability, such risk factors could not be included in this application of the model. Information on the type and biosecurity of farms would further improve our knowledge of the location of farms with varying levels of biosecurity and therefore identify areas more susceptible to outbreaks.

As the model does not currently consider temporal changes in risk factors, future improvements in the model could be achieved by considering seasonality. Timing of influenza transmission among human population, as well as the presence and density of nesting and wintering birds can both be affected by seasonality and different suitability maps could be generated for each season. Traceability data in the context of livestock interfaces would help to understand origins and movements of infected animals throughout value chains, particularly across different types of poultry farms and live animal markets. Finally, future work could investigate viral sequencing information on reported cases to improve our understanding of the linkages between interfaces of wild bird and farmed animals. Such information could help identify routes of entry of influenza into the country through trade or migratory birds and help to narrow the focus of surveillance.

## 5 Conclusions

The development and application of the ZIDAR framework provides an evidence-base SDSS to inform operational decisions on risk-based surveillance strategies and a more efficient allocation of resources to mitigate the impact of zoonotic influenza on wildlife, animal production industry and public health. The process to develop the ZIDAR framework presented here can be adopted by other high-risk countries allowing operational decisions for the control and prevention of zoonotic influenza in their relevant interfaces [28,29], or its application to other diseases with pandemic potential for which risk of exposure spans across wildlife, animal and human populations.

## Funding

This activity was executed with funding support provided through the cooperative agreement between the WHO Regional Office for South-East Asia, New Delhi, and the United States Centers for Disease Control, Atlanta, No. CDC-RFA-IP21-2102: Surveillance and Response to Seasonal and Pandemic Influenza by Regional Offices of the World Health Organization [NU51IP000938].

## CRediT authorship contribution statement

**Adam Charette-Castonguay:** Writing – original draft, Validation, Methodology, Formal analysis, Data curation. **Dipendra Gautam:** Writing – review & editing, Validation, Resources, Project administration, Funding acquisition. **Binay Shrestha:** Writing – review & editing, Validation, Resources, Project administration, Investigation. **Hemant Chandra Ojha:** Validation, Project administration, Investigation. **Barun Kumar Sharma:** Validation, Resources, Project administration, Investigation. **Mukul Upadhayaya:** Validation, Resources, Project administration, Investigation. **Sujan Rana:** Validation, Resources, Project administration, Investigation. **Roshika Shrestha:** Validation, Resources, Project administration, Investigation. **Lok Bandu Chaudhary:** Validation, Resources, Project administration, Investigation. **Bhawana Kandel:** Validation, Resources, Project administration, Investigation. **Rudra Prasad Marasini:** Validation, Resources, Project administration, Investigation. **Sharmila Chapagain:** Validation, Resources, Project administration, Investigation. **Tulsi Ram Gompo:** Validation, Resources, Project administration, Investigation. **Surendra Karki:** Validation, Resources, Project administration, Investigation. **Apsara Poudel:** Validation, Resources, Project administration, Investigation. **Saugat Shrestha:** Validation, Resources, Project administration, Investigation. **Avinash Sunny Kayastha:** Validation, Resources, Project administration, Investigation. **Arun Kumar Govindakarnavar:** Writing – review & editing, Validation, Resources, Project administration, Investigation. **Reuben Samuel:** Validation, Resources, Project administration, Funding acquisition. **Allison Gocotano:** Validation, Resources, Project administration, Funding acquisition. **Pushpa Ranjan Wijesinghe:** Validation, Resources, Project administration, Funding acquisition. **Nilesh Buddha:** Validation, Resources, Project administration, Funding acquisition. **Edwin Ceniza Salvador:** Validation, Resources, Project administration, Funding acquisition. **Manish Kakkar:** Writing – review & editing, Validation, Resources, Project administration, Investigation, Funding acquisition, Conceptualization. **Ricardo J. Soares Magalhães:** Writing – review & editing, Validation, Supervision, Resources, Project administration, Methodology, Conceptualization.

## Declaration of competing interest

The authors declare that they have no known competing financial interests or personal relationships that could have appeared to influence the work reported in this paper.

## Figures and Tables

**Appendix Table 1:** Datasets used for risk factors in the model.

Dataset	Risk factor	Native resolution	Source
Avian Influenza outbreak data	Influenza outbreak period up to the local level and with GPS coordinates	Point location	FAO [35]
Human population	Population density	1 km	WorldPop [36]
Wealth index	Socio-economic status	1 km	Bosco et al. [37]
Livestock population and production	Total number of chicken, ducks and pigs per district (2021)	Polygons	Department of Livestock Services [31]
	Densities of chicken, ducks and pigs per pixel	10 km	FAO [30]
Migratory bird data	Density of migratory bird species	9 km	Ebird [32]
Weather data	Optimal temperature for viral survival	1 km	Fick and Hijmans [38]
Protected areas	Location of protected areas	Polygons	UNEP-WCMC and IUCN [39]
Land cover	Habitat fragmentation	30 m	Provided by Nepalese Forest Research and Training Centre
Waterbodies	Proximity to waterbodies	Polygons	
Wildlife in captivity	Proximity to wildlife sanctuaries	Point location	Provided by Nepal Central Zoo staff
Live markets	Proximity to live animal markets	Point location	Livestock Market Promotion Office [40]
Animal quarantine	Proximity to animal quarantine facilities	Point location	Ministry of Agriculture and Livestock Development [41]

**Appendix Table 2:** Interfaces identified to build the ZIDAR model by local experts.

Interfaces of zoonotic influenza exposure	Average weight (min - max)
Wildlife interfaces	
Migratory bird flyway	0.4 (0.3–0.48)
Poultry/Swine in buffer zone	0.27 (0.17–0.34)
Wildlife captivity settings	0.15 (0.09–0.2)
Areas of habitat fragmentation	0.11 (0.08–0.14)

Poultry interfaces	
Poor farming biosecurity systems	0.42 (0.38–0.49)
Cross-border movements of poultry	0.24 (0.18–0.28)
Live animal markets	0.15 (0.1–0.18)
Mixed poultry farming	0.12 (0.1–0.14)
Farms close to waterbodies	0.07 (0.07–0.08)

Swine interfaces	
Poor biosecurity farming	0.44 (0.4–0.46)
Live animal Markets	0.2 (0.14–0.27)
Cross-border interfaces	0.15 (0.11–0.2)
Integrated farming systems	0.08 (0.06–0.11)

Human interfaces	
Mixed backyard farmers	0.4 (0.31–0.44)
Commercial farmers	0.24 (0.18–0.3)
Butchers and slaughterhouse workers	0.18 (0.14–0.21)
Veterinary professionals	0.12 (0.1–0.17)
Animal traders	0.07 (0.05–0.09)

**Appendix Table 3:** Risk factors for the ZIDAR-W and ZIDAR-A.

Risk factors for zoonotic influenza exposure	Average weight (min - max)
Wildlife risk factors	
Proximity to water bodies	0.2 (0.16–0.22)
Density of migratory bird species	0.15 (0.12–0.19)
Habitat fragmentation	0.13 (0.08–0.18)
Duck density	0.1 (0.05–0.12)
Chicken density	0.08 (0.05–0.11)
Swine density	0.08 (0.06–0.1)
Socioe-conomic status	0.06 (0.03–0.09)
Proximity to wildlife sanctuaries	0.05 (0.05–0.07)

Animal risk factors	
Population density	0.18 (0.16–0.21)
Proximity to water bodies	0.15 (0.11–0.19)
Duck density	0.14 (0.11–0.17)
Chicken density	0.11 (0.1–0.13)
Habitat fragmentation	0.11 (0.1–0.13)
Swine density	0.09 (0.06–0.11)
Optimal temperature for viral survival	0.06 (0.05–0.07)
Socio-economic status	0.06 (0.04–0.07)
Proximity to wildlife sanctuaries	0.05 (0.04–0.06)
Proximity to live animal markets	0.05 (0.04–0.06)
Proximity to animal quarantine facilities	0.04 (0.03–0.06)

**Figure:**
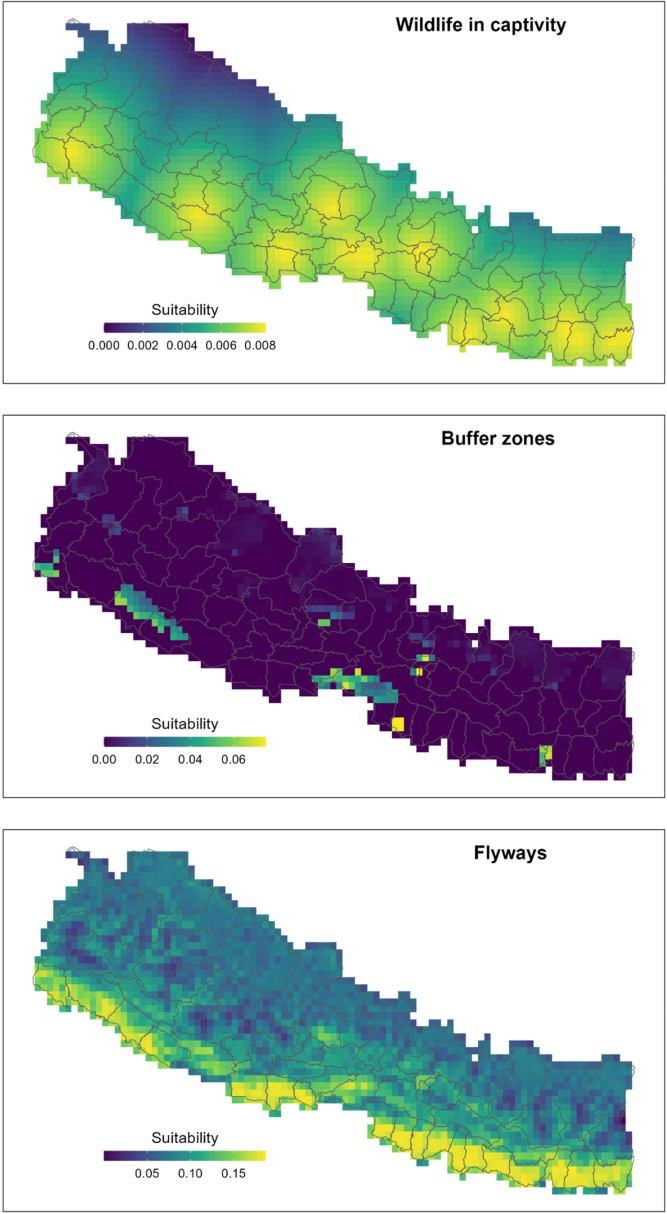

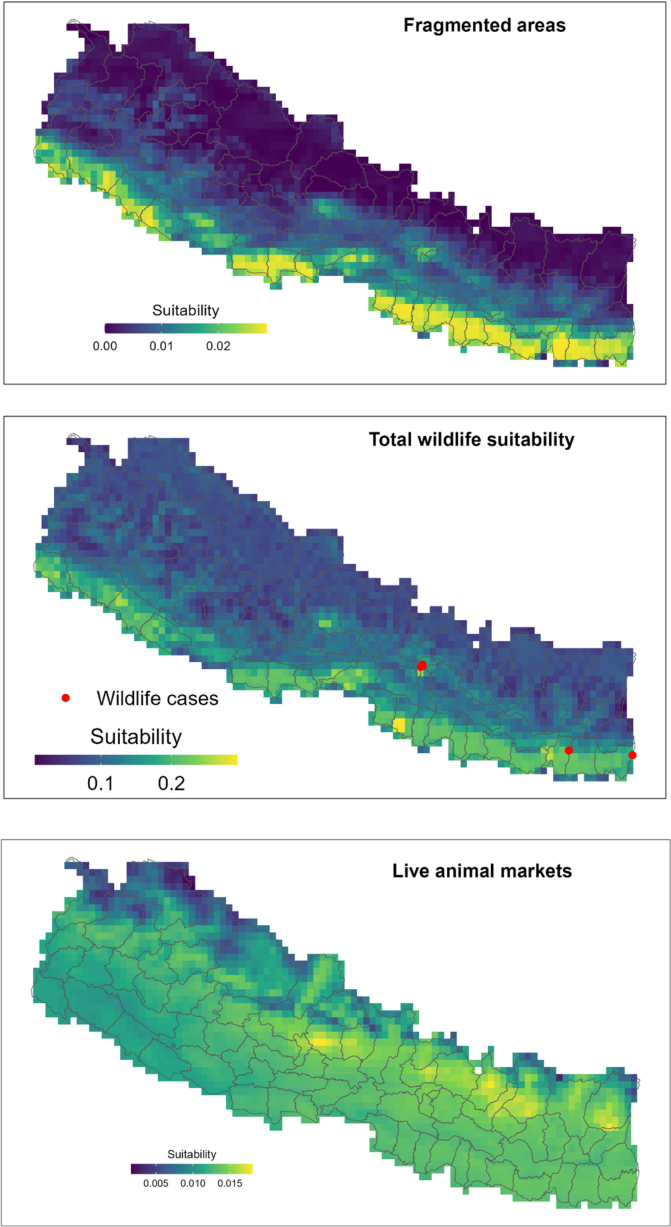

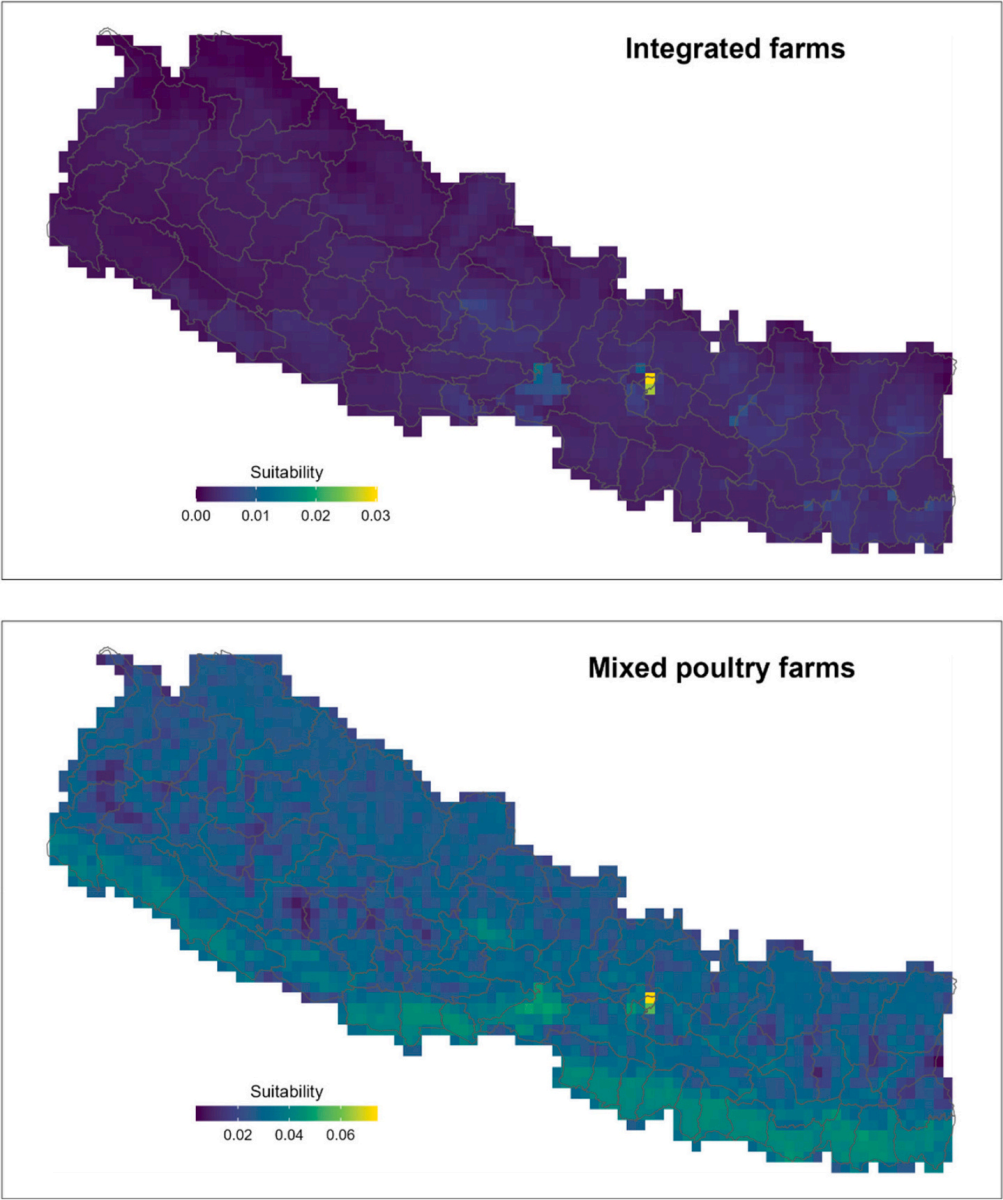

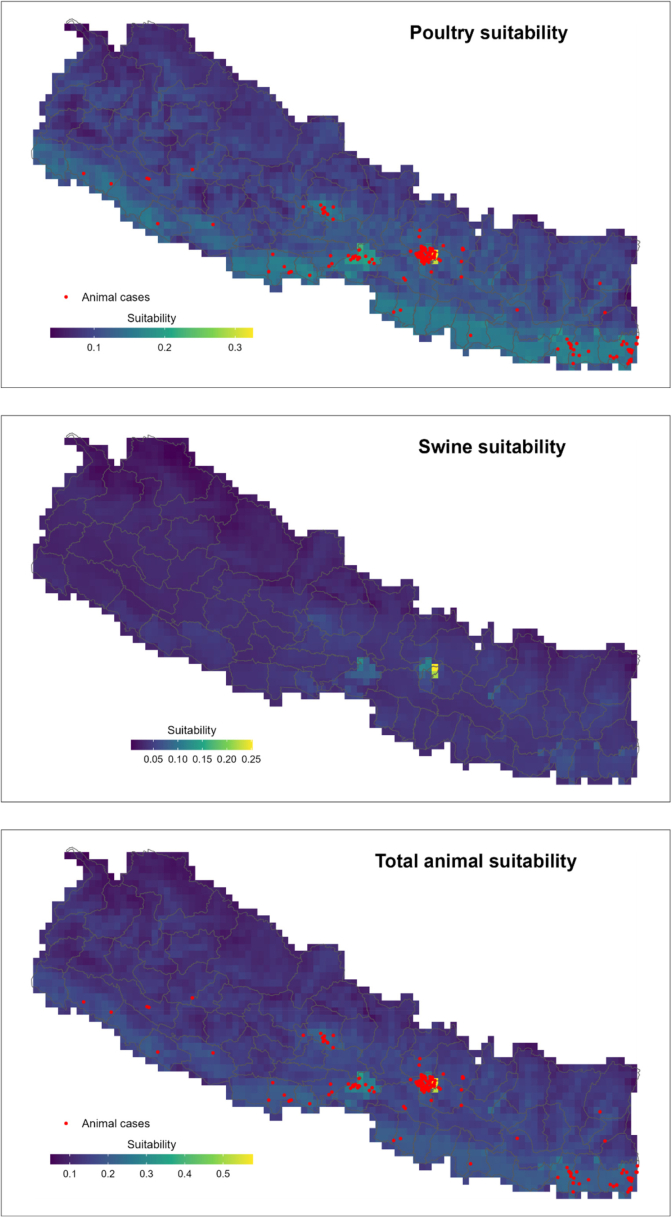

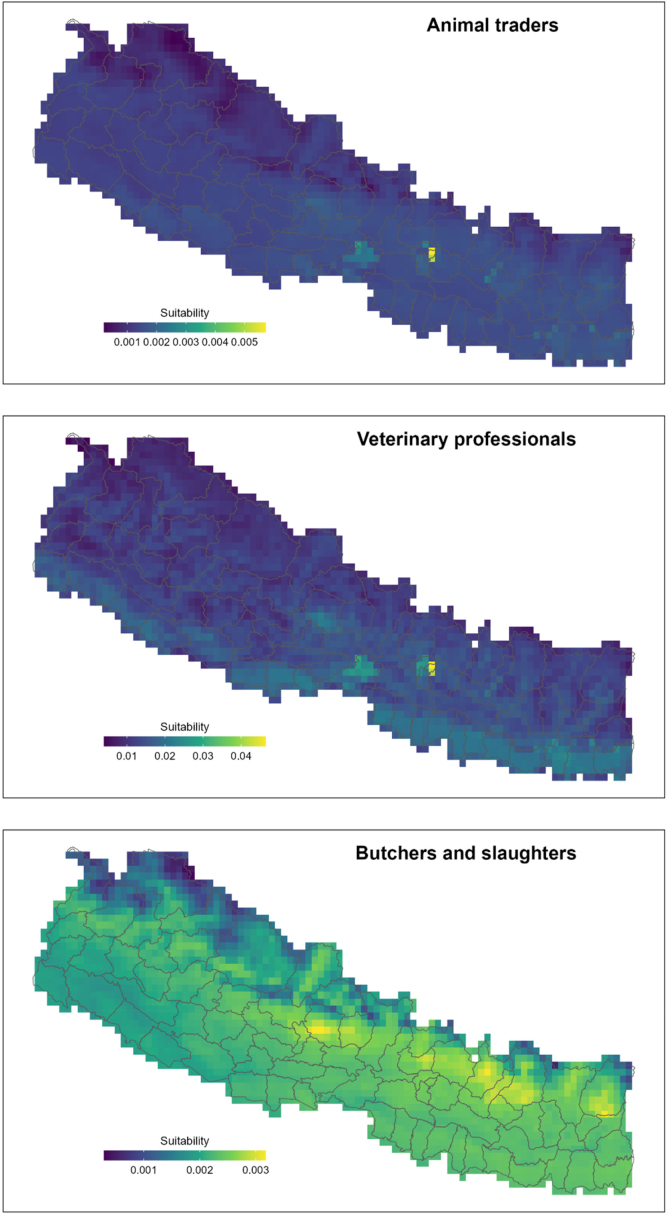

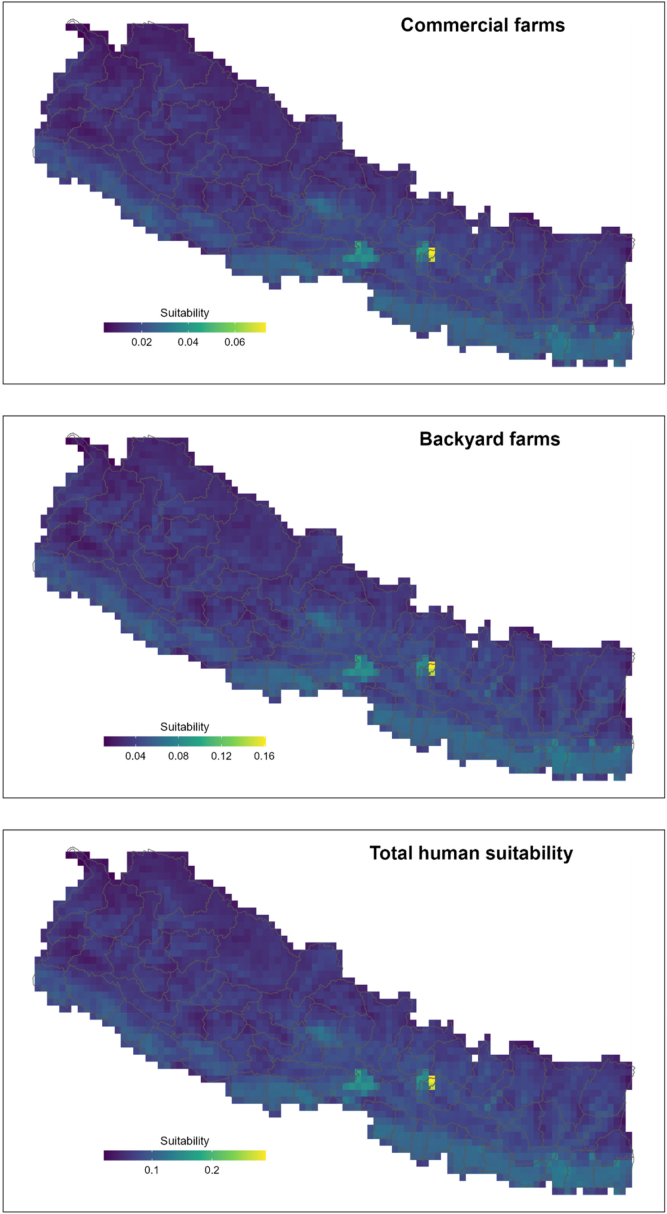


**Fig. A2:**
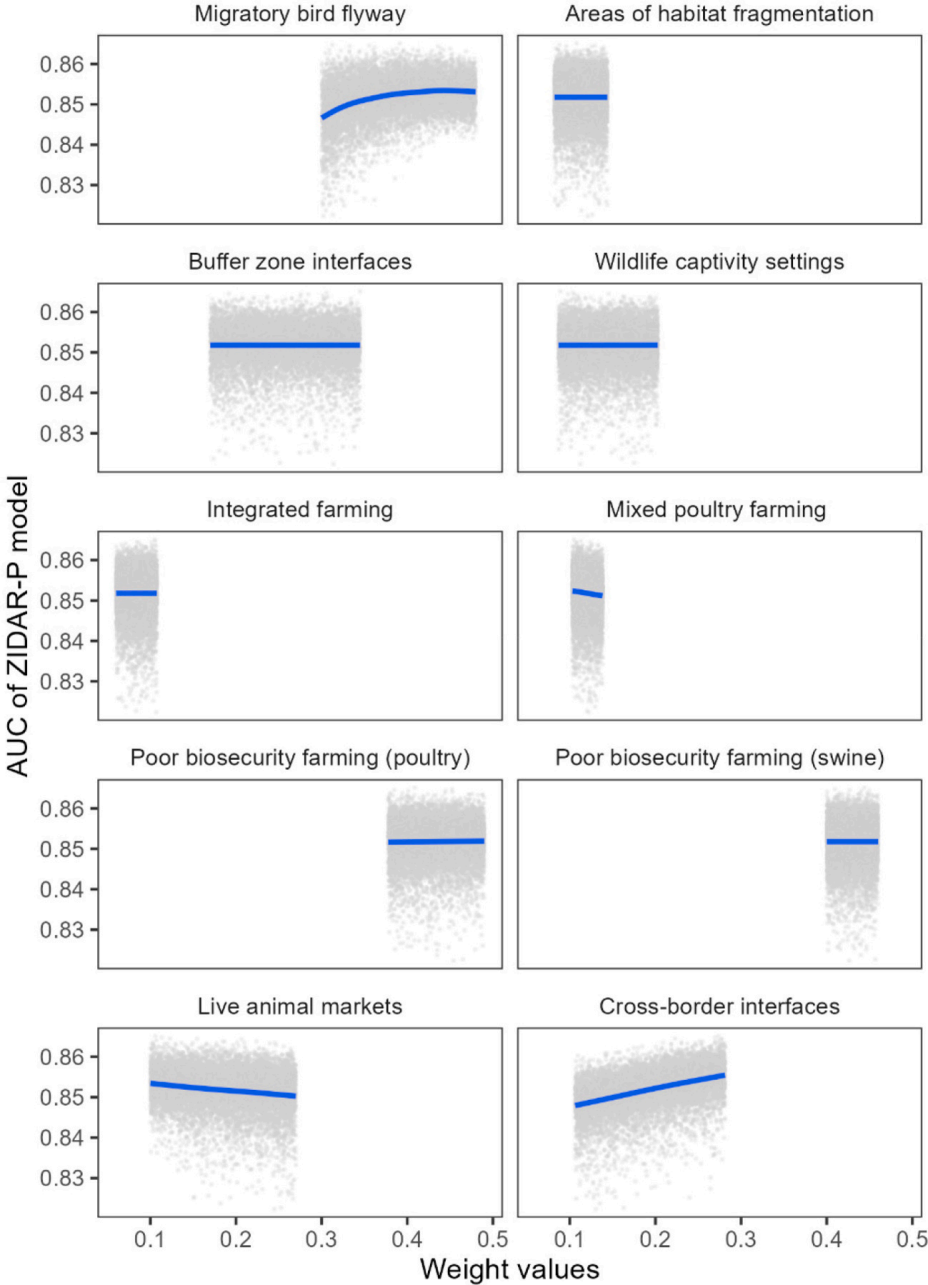
Association between the weight attributed to each transmission interface of ZIDAR-A and the AUC of animal suitability after 10,000 simulation runs. Note that interfaces Wildlife in captivity settings and Poor biosecurity farming (swine) were not part of the model validation of ZIDAR-P.

## Data Availability

Data will be made available on request.

## Appendix A Appendix

### Appendix 1. Expert elicitation workshops in Nepal

A two-day workshop war organised to elicit expert to identify critical interfaces and risk factors for zoonotic influenza exposure risk across human, animal and wildlife populations in Nepal. A total of eight human health experts, eight animal health experts and six environmental health experts from a total of 10 institutions is Nepal participated in the workshop. Experts identified five different human interfaces of exposure, five poultry interfaces, five swine interfaces and five wildlife interfaces, for a total of 20 interfaces. In addition, experts identified eight risk factors for human, 13 for poultry, 14 for swine and 11 for wildlife interfaces, for a total of 46 risk factors. A second workshop with the same experts was later organised to co-design the conceptual model and directed acyclic graphs including interfaces and risk factors. Given data availability the number of interfaces was later reduced to 18 (Appendix Table 2) and the number of risk factors to 19 (Appendix Table 3) during the second workshop.

## Appendix 2. Model parameters for Nepal application

Datasets used as inputs in the model are shown in Appendix Table 1. Due to inconsistencies between the Gridded Livestock of the World and official census information, livestock densities (chicken, duck and pig) per pixel were recalculated based on the fraction of animals on a pixel of the total animals in a district [30] and the total animals in a district [31]. We estimated the density of migratory bird species by aggregating occurrence of 56 species of migratory and water birds [32] on each pixel. Optimal temperature for viral survival was estimated by calculating the number of months with average temperature between 5 and 16 degrees for each pixel given the higher efficiency of transmission under colder conditions [33]. To estimate habitat fragmentation, we used the fraction of a pixel under urban and cropland cover from Landsat land cover classification provided by the Nepalese Forest Research and Training Centre. Proximity to water bodies and wildlife sanctuaries were calculated using the Euclidean distance from a pixel to the nearest feature. Proximity to live animal markets and animal quarantine facilities were estimated by calculating travel time from a pixel to the nearest feature based on the friction surface produced by Weiss et al. [34].

## References

[1] The Global Consortium for H5N8 and Related Influenza Viruses (2016). Role for migratory wild birds in the global spread of avian influenza H5N8. Science.

[2] Watanabe Y., Ibrahim M.S., Suzuki Y., Ikuta K. (2012). The changing nature of avian influenza a virus (H5N1). Trends Microbiol..

[3] Bloom E.A., De Wit V., Jose C.-S. (2005).

[4] Farahat R.A., Khan S.H., Rabaan A.A., Al-Tawfiq J.A. (2023). The resurgence of avian influenza and human infection: a brief outlook. New Microbes New Infect..

[5] Bui C.M., Gardner L., MacIntyre R., Sarkar S. (2017). Influenza a H5N1 and H7N9 in China: a spatial risk analysis. PLoS One.

[6] Fang L.-Q., de Vlas S.J., Liang S., Looman C.W.N., Gong P., Xu B., Yan L., Yang H., Richardus J.H., Cao W.-C. (2008). Environmental factors contributing to the spread of H5N1 avian influenza in mainland China. PLoS One.

[7] Gilbert M., Xiao X., Pfeiffer D.U., Epprecht M., Boles S., Czarnecki C., Chaitaweesub P., Kalpravidh W., Minh P.Q., Otte M.J., Martin V., Slingenbergh J. (2008). Mapping H5N1 highly pathogenic avian influenza risk in Southeast Asia. Proc. Natl. Acad. Sci..

[8] Pfeiffer D.U., Minh P.Q., Martin V., Epprecht M., Otte M.J. (2007). An analysis of the spatial and temporal patterns of highly pathogenic avian influenza occurrence in Vietnam using national surveillance data. Vet. J..

[9] La Sala L.F., Burgos J.M., Blanco D.E., Stevens K.B., Fernández A.R., Capobianco G., Tohmé F., Pérez A.M. (2019). Spatial modelling for low pathogenicity avian influenza virus at the interface of wild birds and backyard poultry. Transbound. Emerg. Dis..

[10] Paul M.C., Goutard F.L., Roulleau F., Holl D., Thanapongtharm W., Roger F.L., Tran A. (2016). Quantitative assessment of a spatial multicriteria model for highly pathogenic avian influenza H5N1 in Thailand, and application in Cambodia. Sci. Rep..

[11] Stevens K.B., Gilbert M., Pfeiffer D.U. (2013). Modeling habitat suitability for occurrence of highly pathogenic avian influenza virus H5N1 in domestic poultry in Asia: a spatial multicriteria decision analysis approach. Spat. Spatio-tempor. Epidemiol..

[12] Prosser D.J., Hungerford L.L., Erwin R.M., Ottinger M.A., Takekawa J.Y., Newman S.H., Xiao X., Ellis E.C. (2016). Spatial modeling of wild Bird risk factors for highly pathogenic a(H5N1) avian influenza virus transmission. Avian Dis..

[13] Hassan O.A., de Balogh K., Winkler A.S. (2023). One health early warning and response system for zoonotic diseases outbreaks: emphasis on the involvement of grassroots actors. Veterin. Med. Sci..

[14] Zhang X.-X., Lederman Z., Han L.-F., Schurer J.M., Xiao L.-H., Zhang Z.-B., Chen Q.-L., Pfeiffer D., Ward M.P., Sripa B., Gabriël S., Dhama K., Acharya K.P., Robertson L.J., Deem S.L., Aenishaenslin C., Dantas-Torres F., Otranto D., Grace D., Wang Y., Li P., Fu C., Poeta P., Tanvir Rahman Md., Kassegne K., Zhu Y.-Z., Yin K., Liu J., Wang Z.-J., Guo X.-K., Gong W.-F., Schwartländer B., Ren M.-H., Zhou X.-N. (2024). Towards an actionable one health approach. Infect. Dis. Poverty.

[15] Karmacharya D., Manandhar S., Sharma A., Bhatta T., Adhikari P., Sherchan A.M., Shrestha B., Bista M., Rajbhandari R., Oberoi M., Bisht K., Hero J.-M., Dissanayake R., Dhakal M., Hughes J., Debnath N. (2015). Surveillance of influenza a virus and its subtypes in migratory wild birds of Nepal. PLoS One.

[16] Palm E.C., Newman S.H., Prosser D.J., Xiao X., Ze L., Batbayar N., Balachandran S., Takekawa J.Y. (2015). Mapping migratory flyways in Asia using dynamic Brownian bridge movement models. Movem. Ecol..

[17] Takekawa J.Y., Palm E.C., Prosser D.J., Hawkes L.A., Batbayar N., Balachandran S., Luo Z., Xiao X., Newman S.H., Prins H.H.T., Namgail T. (2017). Bird Migration across the Himalayas: Wetland Functioning Amidst Mountains and Glaciers.

[18] Department of Livestock Services (2008).

[19] Poudel U., Dahal U., Upadhyaya N., Chaudhari S., Dhakal S. (2020). Livestock and poultry production in Nepal and current status of vaccine development. Vaccines (Basel).

[20] Saaty T.L. (1980).

[21] World Organisation for Animal Health (OIE). (n.d.). World Animal Health Information System (WAHIS). Accessed [August/2023], https://www.woah.org/en/what-we-do/animal-health-and-welfare/disease-data-collection/world-animal-health-information-system/.from.

[22] Roocks P. (2016). Computing Pareto frontiers and database preferences with the rPref package. R J..

[23] Fisher W.D. (1958). On grouping for maximum homogeneity. J. Am. Stat. Assoc..

[24] Bivand R., Ono H., Dunlap R., Stigler M., Bivand M.R. (2015).

[25] Larkins A., Bruce M., Phetsouvanh R., Ash A. (2023). Risk mapping for Taenia solium: applying multicriteria decision analysis in Lao PDR. Trop. Med. Int. Health.

[26] Rey S.J., Stephens P., Laura J. (2017). An evaluation of sampling and full enumeration strategies for Fisher Jenks classification in big data settings. Trans. GIS.

[27] Karki S., Lupiani B., Budke C.M., Karki N.P., Rushton J., Ivanek R. (2015). Cost-benefit analysis of avian influenza control in Nepal. Rev. Sci. Tech..

[28] Krammer F., Schultz-Cherry S. (2023). We need to keep an eye on avian influenza. Nat. Rev. Immunol..

[29] Verhagen J.H., Fouchier R.A.M., Lewis N. (2021). Highly Pathogenic Avian Influenza Viruses at the Wild–Domestic Bird Interface in Europe: Future Directions for Research and Surveillance. Viruses.

[30] FAO (2022).

[31] Department of Livestock Services (2021).

[32] Ebird (2017).

[33] Lowen A.C., Steel J. (2014). Roles of humidity and temperature in shaping influenza seasonality. J. Virol..

[34] Weiss D.J., Nelson A., Gibson H.S., Temperley W., Peedell S., Lieber A., Hancher M., Poyart E., Belchior S., Fullman N., Mappin B., Dalrymple U., Rozier J., Lucas T.C.D., Howes R.E., Tusting L.S., Kang S.Y., Cameron E., Bisanzio D., Battle K.E., Bhatt S., Gething P.W. (2018). A global map of travel time to cities to assess inequalities in accessibility in 2015. Nature.

[35] FAO (2023). EMPRES-I: Avian Influenza Reported Cases. https://www.who.int/tools/flunet.

[36] WorldPop (2018). Funded by The Bill Melinda Gates Foundation (OPP1134076) Sch. Geogr. Environ. Sci. Univ. Southampton.

[37] Bosco C., Tejedor-Garavito N., de Rigo D., Tatem A.J., Pezzulo C., Wood R., Chamberlain H., Bird T. (2018).

[38] Fick S.E., Hijmans R.J. (2017). WorldClim 2: new 1km spatial resolution climate surfaces for global land areas. Int. J. Climatol..

[39] UNEP-WCMC and IUCN (2023). Protected Planet: The World Database on Protected Areas (WDPA). http://www.protectedplanet.net.

[40] Livestock Market Promotion Office (2018).

[41] Ministry of Agriculture and Livestock Development (2022). Animal Quarantine facilities- Krishi Diary. http://aitc.gov.np/english/downloadsdetail/2/2019/19794382/.

